# Sweet grass protection against oxidative stress formation in the rat brain

**DOI:** 10.1007/s11011-014-9599-z

**Published:** 2014-08-10

**Authors:** Wojciech Łuczaj, Iwona Jarocka-Karpowicz, Katarzyna Bielawska, Elżbieta Skrzydlewska

**Affiliations:** Medical University of Białystok, Białystok, Poland

**Keywords:** Coumarins, Oxidative stress, Ethanol, Lipid peroxidation, Antioxidants, Brain

## Abstract

The aims of this study were to investigate the influences of sweet grass on chronic ethanol-induced oxidative stress in the rat brain. Chronic ethanol intoxication decreased activities and antioxidant levels resulting in enhanced lipid peroxidation. Administration of sweet grass solution to ethanol-intoxicated rats partially normalized the activity activities of Cu,Zn-superoxide dismutase, catalase, glutathione peroxidase, and glutathione reductase, as well as levels of reduced glutathione and vitamins C, E, and A. Sweet grass also protected unsaturated fatty acids (arachidonic and docosahexaenoic) from oxidations and decreased levels of lipid peroxidation products: 4-hydroxynonenal, isoprostanes, and neuroprostanes. The present in vivo study confirms previous in vitro data demonstrating the bioactivity of sweet grass and suggests a possible role for sweet grass in human health protection from deleterious consequences associated with oxidative stress formation.

## Introduction

Cells in the nervous systems of animals, including humans, are vulnerable to oxidative damage from reactive oxygen species (ROS). Reasons for this vulnerability include a high concentration of readily oxidizable substrates (especially membrane phospholipid polyunsaturated fatty acids), a high ratio of membrane surface area to cytoplasmic volume, and an extended axonal morphology that is prone to peripheral injury and low levels of protective antioxidant enzymes (e.g., catalase and glutathione [GSH] peroxidase [GSH-Px]) (Enciu et al. 2013; Kerman et al. 2005). A large amount of oxygen is also consumed in a relatively small tissue mass, and some brain regions have high nonheme iron concentrations (Shila et al. 2005).

Moreover, many brain mechanisms promote ROS generation (Joseph et al. 2005). For example, the oxidation of neurotransmitters is accompanied by ROS generation. Activated microglia, as the resident macrophages of the nervous system, can also participate in ROS generation. Thus, antioxidative defense is critically important in nervous tissue protection. If mild oxidative stress occurs, normal tissues often respond by bolstering antioxidant defense, although severe or persistent oxidative stress can cause cellular component injury, cellular degeneration, and, finally, brain cell death (Venkataraman et al. 2010).

Chronic ethanol consumption induces free-radicals production and consequent oxidative damage (Pronko et al. 2002; Koop 2006). Due to ethanol metabolism, the central nervous system (CNS) is influenced by substantial amounts of superoxide anion as the first ROS (Jung et al. 2008). Superoxide dismutase metabolizes superoxide anion into hydrogen peroxide, which can activate some neurotransmitters responsible for control such as dopamine (Adachi et al. 2001). Under the influence of iron (II) ions hydrogen peroxide is converted to the highly reactive hydroxyl radical, which can react with ethanol to form hydroxyethyl radicals (Sadrzadeh and Saffari 2004). In the CNS, the hydroxyethyl radical level is increased by isoenzyme CYP2E1 induction after ethanol intoxication (Sun and Sun 2001). Hydroxyethyl radicals react with acetaldehyde, an ethanol metabolite, resulting in increased CNS levels of acetyl radicals (Castro et al. 2009).

Potent antioxidants, especially those belonging to natural products, are under investigation for their potential ability to prevent oxidative stress and its consequences. One such potentially health-promoting plant is sweet grass (*Hierochloë odorata*), which belongs to the *Graminacae* grass family. Sweet grass is a strongly aromatic perennial grass normally found growing in the rich, moist soils of North America, Asia, and Europe. Although its chemical composition and biological properties have not been extensively investigated, it contains, among other compounds, coumarin and its derivatives 5,8-dihydroxycoumarin and 5-hydroxy-8-O-β-D-glucopyranosyl coumarin (Grigonis et al. 2005). Coumarin hydroxyl derivatives have been reported to have antioxidative and health-promoting properties (Kostova, 2006; Thuong et al. 2010; Li et al. 2013).

Previously Łuczaj et al. (2012) showed that a sweet grass beverage partially protects the liver against ethanol oxidative stress. Evidence of its antioxidative action is still growing, with the strong free radical scavenging and antioxidant properties of 5,8-dihydroxycoumarin confirmed in a recent study (Slapšytė et al. 2013). Therefore, the aim of this study was to investigate the influence of the consumption of a sweet grass beverage on oxidative stress formation and consequences in the brains of rats intoxicated with ethanol.

## Materials and methods

Sweet grass extract used in the experiment contained coumarin (312 mg/l), 5,8-dihydroxycoumarin (4,2 mg/l) and 5-hydroxy-8-O-β -D-glucopyranosyl-benzopyranone (3,1 mg/l). The level of these compounds was determined using a gas chromatograph (Agilent Technologies) equipped with a triple quadrupole detector in the electron-impact ionization mode (GC System 7890A with GC/MS Triple Quad 7,000) and HP-5MS capillary column (30 m × 0.25 mm, ID 0.2 μm, Agilent Technologies). The system was equipped with autosampler (Agilent Technologies G4513A). Instrument control and data analysis were performed with Agilent GC software, MassHunter B.06.00. The column temperature was initially set at 50 °C, and then raised at 10 °C/min to 280 °C and maintained at this temperature for 10 min. The split-splitless injector was used in split mode with a split ratio of 1:20. The carrier gas was helium at a flow rate of 1 mL/min. The injector and the transfer line were kept at 280 °C, and the source temperature was set at 230 °C. The MS unit was operated in scan mode (50–500 m/z). The coumarin peak was identified by comparison of the retention time with the standard and its mass spectrum by using the National Institute of Standards and Technology Virtual Library (NIST) and the 5,8-dihydroxycoumarin and the 5-hydroxy-8-O-β -D-glucopyranosyl-benzopyranone were identified by comparison with theirs mass spectrum. The concentration of coumarins in the sample was calculated using an external standard method (1–500 μg/ml, R^2^ = 0,9982).

### Animals

12 months old male Wistar rats were used for the experiment. They were housed in groups with free access to a granular standard diet and water and maintained under a normal light–dark cycle. The rats were weighed every week of experiment and changes in the weight of animals from different groups were not statistically significant. All experiments were approved by the Local Ethic Committee in Białystok (Poland) referring to Polish Act Protecting Animals of 1997.

The animals were divided into the following groups:

- Control group. Rats were treated intragastrically with 1.8 ml of physiological saline every day for 4 weeks (*n* = 6).
- Sweet grass group. Rats received sweet grass water beverage (coumarin content 10 mg/l of water) ad libitum instead of water for 1 week. Next they were treated intragastrically with 1.8 ml of physiological saline and received sweet grass water beverage ad libitum instead of water every day for 4 weeks (*n* = 6).
- Ethanol group. Rats were treated intragastrically with 1.8 ml of ethanol in doses from 2.0 to 6.0 g/kg b.w. every day for 4 weeks. The dose of ethanol was gradually increased by 0.5 g/kg b.w. every 3 days (*n* = 6).
- Sweet grass and ethanol group. Rats received sweet grass water beverage ad libitum instead of water for 1 week. Next they were treated intragastrically with 1.8 ml of ethanol in doses from 2.0 to 6.0 g/kg b.w. and received sweet grass water beverage ad libitum instead of water every day for 4 weeks.

### Preparation of tissue

After the above procedure, the rats were sacrified under ether anaesthesia (six animals in each group). Brain was removed quickly and placed in iced 0.15 M NaCl solution, washed with the same solution to remove blood cells, blotted on filter paper, weighed and homogenied in 9 ml ice-cold 0.25 M sucrose and 0.15 M NaCl with the addition of 6 μl 250 mM BHT (butylated hydroxytoluene) in ethanol to prevent the formation of new peroxides during the assay. Homogenization procedure was performed under standardized conditions; 10 % homogenates were centrifuged at 10.000 × g for 15 min at 4 °C, and the supernatant was kept on ice until assayed.

### Biochemical assays

Superoxide dismutase (Cu,Zn–SOD – EC.1.15.1.1) activity was determined by the method of Misra and Fridovich (1972) as modified by Sykes et al. (1978), which measures the activity of cytosolic SOD. Mn–SOD of the brain mitochondria is known to be destroyed during this procedure. A standard curve for SOD activity was constructed using SOD from bovine erythrocytes (Sigma Biochemicals St. Louis MO). One unit of SOD was defined as the amount of the enzyme, which inhibits epinephrine oxidation to adrenochrome by 50 %. The enzyme specific activity was expressed in units per mg of protein.

Catalase (CAT – EC.1.11.1.9) activity was determined after 30 min preincubation of the postmitochondrial fraction of the brain homogenate with 1 % Triton X-100 by measuring the decrease in absorbance of hydrogen peroxide at 240 nm (Aebi, 1984). The rates were determined at 25 °C using 10 mM hydrogen peroxide and the activity was expressed in units per mg of protein. One unit of CAT was defined as the amount of the enzyme required to catalyze 1 μmol H_2_O_2_ during 1 min. The enzyme specific activity was expressed in units per mg of protein.

Glutathione peroxidase (GSH-Px – EC.1.11.1.6) activity was assessed in the brain spectrophotometrically using a technique based on Paglia and Valentine (1967). Applying this technique, GSH formation was assayed by measuring the conversion of nicotinamide adenine dinucleotide phosphate (NADPH) to NADP. The final concentration of GSH was 0.2 mM and of H_2_O_2_ was 0.3 mM. The activity was expressed in universal units. One unit of activity was defined as the amount of enzyme catalyzing the oxidation of 1 μmol of NADPH min^−1^, at 25 °C and pH 7.4. The enzyme specific activity was expressed in units per mg of protein.

Glutathione reductase (GSSG-R – EC.1.6.4.2) activity was measured by the method of Mize and Langdon (1962) by monitoring the oxidation of nicotinamide adenine dinucleotide phosphate (NADPH) at 340 nm. The reaction mixture contained 0.2 mM KCl, 1 mM EDTA and 1 mM oxidized GSH (GSSG) in 0.1 M potassium phosphate buffer, pH 7.1. The enzyme activity was expressed in units per mg of protein. One unit of GR oxidized 1 mmol of NADPH/min at 25°C and pH 7.4. The enzyme specific activity was expressed in units per mg of protein.

GSH level was measured using the GSH/GSSG kit (Alpco Diagnostics, Salem, NH) according to kit instructions. The kit provides the HPLC method with fluorescence detection (λ_Ex_ = 385 nm, λ_Em_ = 515 nm; isocratic elution; flow rate: 1 ml/min.).

The HPLC methods were used to determine the level of vitamin C (Ivanovic et al. 1999), vitamin A and E (De Leenher et al. 1979) and β-carotene (Elinder, 1992). To determine ascorbic acid 300 μl of brain homogenate was mixed with an equal volume of metaphosphoric acid (100 g/l). Before analysis, the samples were centrifuged (3,500 × g, 4 min) to remove precipitated protein after which they were immediately assayed. The vitamin A and E were extracted from brain homogenate with hexane containing 0.025 % butylated hydroxytoluene. The hexane phase was removed and dried with sodium sulfate, and 50 μl of the hexane extract was injected on the column.

Free and phospholipid arachidonic acid (AA) and docosahexaenoic acid (DHA) were determined by gas chromatography (Christie 1993). Lipids components were isolated by Folch extraction using chloroform/methanol mixture (2:1, v/v). Using TLC free fatty acids and total phospholipids were separated with the mobile phase heptane - diisopropyl ether – acetic acid (60:40:3, v/v/v). All lipid fractions were transmetylated to fatty acid methyl esters (FAMEs) with boron trifluoride in methanol reagent under nitrogen atmosphere without previous separation from the layer. The FAME’s were analyzed by gas chromatography. The system consisted of a Perkin Elmer Clarus 500 gas chromatograph with FID (flame ionization detector) equipped with a Perkin Elmer AS XL autosampler. Separation of FAME was carried out on capillary column coated with Varian CP-Sil88 stationary phase.

Lipid peroxidation was estimated by measuring of 4-hydroxynonenal (4–HNE), F_2_-isoprostanes (8-isoPGF_2α_) and total neuroprostanes level. 4-HNE was determined as a one of the fluorescent decahydroacridine derivatives formed in reaction with 1,3-cyclohexanodione. It was determined by HPLC with fluorescence detector with excitation at 380 nm and emission at 445 nm (Yoshino et al. 1986). Total brain F_2_-isoprostanes (8-isoPGF_2α_) and A_4_/J_4_-neuroprostanes (NPs) were quantified using modified high performance liquid chromatography mass spectrometry methods of Coolen and Fam respectively (Coolen et al. 2005 and Fam et al. 2002). 8-isoPGF_2α_ as well as NPs were isolated using SPE method, after an alkaline hydrolysis step. All analysis were performed using an Agilent 1290 UPLC system interfaced with an Agilent 6,460 triple quadrupole mass spectrometer with electrospray ionization source (ESI). The system was equipped with autosampler (Agilent Technologies G4513A) and autosampler thermostat (Agilent Technologies G1330B). Instrument control and data analysis were performed with Agilent LC MassHunte software, rev. B.05.00. A Zorbax Eclipse Plus C18 (2.1 mm × 150 mm, 3.5 μm, Agilent, Santa Clara, CA) was employed. The separation was carried out with a linear gradient with water (adjusted to pH 5.7 with acetic acid) and acetonitrile. In both cases tetradeuterated 8-isoPGF_2α_ (8-isoPGF_2α_–d_4_) as an internal standard was used. 8-isoPGF_2α_ was analyzed in negative-ion mode using multiple reaction monitoring (MRM). Transitions of the precursors to the product ions were as follows: *m*/*z* 353.2 → 193.1 (for 8-isoPGF_2α_) and 357.2 → 197.1 (for 8-isoPGF_2α_-d_4_ detection). The concentration of 8-isoPGF_2α_ isomer in the samples was calculated using a calibration curve (1–1,000 pg/ml R^2^ = 0,9,975). NPs were analyzed by selected ion monitoring (SIM) in the m/z 357, as a series of peaks that have molecular masses and retention times expected for NPs generated from the oxidation of DHA in vitro. DHA was oxidized in vitro using an iron/ADP/ascorbate mixture, as described elsewhere.^31^ The pattern of peaks representing A- and J-ring NPs was very similar to that obtained from the oxidation of DHA in vitro*.*

### Statistical analysis

Data obtained in the current study are expressed as mean ± SD. These data were analyzed by using standard statistical analyses, one-way analysis of variance (ANOVA) with Tukey test for multiple comparisons, to determine significant differences between different groups. A *p* value of < 0.05 was considered significant.

## Results

The activity of antioxidant enzymes in the brain was altered by chronic ethanol intoxication (Table 1). After ethanol intoxication, it was showed a significant decrease in the brain activity of antioxidant enzymes such as superoxide dismutase (by about 60 %), GSH-Px (by about 24 %), GSSG-R (by about 37 %), and catalase (by about 37 %) compared to the control. After administration of sweet grass to ethanol intoxicated rats, activities of superoxide dismutase and GSSG-R were similar to the values observed in the brain of control animals. However activities of brain GSH-Px and catalase were significantly decreased (by approximately 10 % and 21 %), compared to the control.

**Table 1:** Antioxidant parameters in the brain of rats chronically intoxicated with ethanol and rats drinking sweet grass beverage and chronically intoxicated with ethanol

Analyzed parameter	Groups of rats			
	Control	Sweet grass	Alcohol	Alcohol + sweet grass
Cu,Zn–SOD (U/mg protein) GSH-Px (U/mg protein) GSSG-R (U/mg protein) CAT (U/mg protein)	55.7 ± 3.2 52.4 ± 2.1 3.51 ± 0.17 0.57 ± 0.02	53.4 ± 3.8 47.3 ± 2.5^a^ 3.28 ± 0.21 0.45 ± 0.02^a^	22.6 ± 2.2^ab^ 39.8 ± 3.5^ab^ 2.20 ± 0.15^ab^ 0.36 ± 0.03^ab^	34.7 ± 2.9^abc^ 45.6 ± 3.32^ac^ 2.95 ± 0.25^abc^ 0.45 ± 0.03^ac^
GSH (μmol/g tissue) Vitamin C (μmol/g tissue) Vitamin E (nmol/g tissue) Vitamin A (nmol/g tissue)	0.95 ± 0.06 2.34 ± 0.16 24.32 ± 1.33 0.42 ± 0.02	0.89 ± 0.07 2.40 ± 0.18 24.95 ± 1.67 0.40 ± 0.02	0.56 ± 0.04^ab^ 1.77 ± 0.13^ab^ 20.12 ± 1.51^ab^ 0.26 ± 0.02^ab^	0.81 ± 0.07^ac^ 2.08 ± 0.16 ^abc^ 22.51 ± 1.67^bc^ 0.33 ± 0.02^abc^

Data points represent mean ± SD; *n* = 6; (^a^ *p* < 0,05 in comparison with control group; ^b^ *p* < 0,05 in comparison with sweet grass group; ^c^ *p* < 0,05 in comparison with alcohol group)

Ethanol intoxication also decreased the levels of nonenzymatic antioxidants, such as GSH (approximately 41 %), and vitamins C, E, and A (approximately 24 %, 17 %, and 38 %, respectively). Sweet grass solution partially prevented changes in the levels of these compounds. Vitamin E level in the brains of ethanol-intoxicated rats that received sweet grass solution remained similar to the control, while the levels of other nonenzymatic antioxidants were still significantly reduced compared to the control group.

**Fig. 1:**
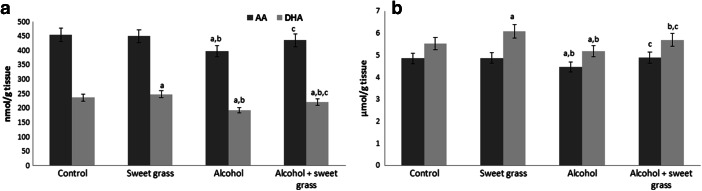
The level of **a** free and **b** phospholipid arachidonic (AA) and docosahexaenoic acids (DHA) in the brain of rats chronically intoxicated with ethanol and rats drinking sweet grass beverage and chronically intoxicated with ethanol. Data points represent mean ± SD; *n* = 6; (^a^ *p* < 0,05 in comparison with control group;^b^ *p* < 0,05 in comparison with sweet grass group; ^c^ *p* < 0,05 in comparison with alcohol group)

Changes in the activities and levels of antioxidants after ethanol intoxication led to enhanced lipid peroxidation in the rat brain, as manifested by significant increases in the levels of lipid peroxidation products, such as 4-hydroxynonenal, and 8-iso-PGF_2α_ (approximately 42 % and 17 %, respectively) compared to the control group (Table 2). The most dramatic change was observed in the level of neuroprostanes (NPs). Increase in NPs after ethanol ingestion were more significant (almost two times higher) than 8-isoPGF_2α_ which is a less specific marker of oxidative brain injury. Administration of sweet grass solution significantly decreased the levels of these markers, especially 8-iso-PGF_2α_ and NPs (approximately 15 % and 11 % respectively). Chronic ethanol consumption influenced the levels of free and phospholipid fatty acids, particularly polyunsaturated fatty acids (PUFAs), corresponding with the increase in lipid peroxidation products observed in this study. Chronic ethanol consumption significantly decreased the levels of free arachidonic acid (AA) and docosahexaenoic acid (DHA) by about 12 % and 19 %, respectively. Drinking the sweet grass solution significantly increased the DHA level (approximately 5 %), but did not cause considerable changes in the AA level (Fig. 1). Administration of sweet grass together with alcohol prevented the decrease in the AA and DHA levels, compared to the ethanol group. After ethanol administration, the phospholipid AA and DHA levels in the brain were decreased by about 8 % and 6 %, respectively. Consumption of sweet grass solution alone caused approximately 10 % increase in the DHA level, but did not cause significant changes in the AA level. Sweet grass given with alcohol increased the levels of AA and DHA (approximately 10 %) compared with the alcohol alone group.

**Table 2:** Lipid peroxidation products (4-HNE, 8-isoPGF_2α,_ and NPs) in the brain of rats chronically intoxicated with ethanol and rats drinking sweet grass beverage and chronically intoxicated with ethanol

Analyzed parameter	Groups of rats			
	Control	Sweet grass	Alcohol	Alcohol + sweet grass
4-HNE (nmol/g tissue) 8-isoPGF_2α_ (ng/g tissue) NPs (ng/g tissue)	1.54 ± 0.09 1.91 ± 0.10 7.38 ± 0.41	1.36 ± 0.10^a^ 1.63 ± 0.09^a^ 6.53 ± 0.33^a^	2.18 ± 0.17^ab^ 2.24 ± 0.12^ab^ 16.69 ± 0.87^ab^	1.84 ± 0.14^abc^ 2.17 ± 0.11^ab^ 13.34 ± 0.69^abc^

Data points represent mean ± SD; *n* = 6; (^a^ *p* < 0,05 in comparison with control group; ^b^ *p* < 0,05 in comparison with sweet grass group; ^c^ *p* < 0,05 in comparison with alcohol group)

## Discussion

In the current study, we investigated whether administration of sweet grass ameliorated the oxidative damage caused to rat brain by ethanol intoxication, because despite the protection offered by the blood–brain barrier, the CNS is subjected to ethanol-associated pathology as a result of ethanol metabolism-induced free radicals (Ponnappa and Rubin 2000, Gonthier et al. 2012). Ethanol oxidation by aldehyde dehydrogenase (ADH) is accompanied by an increase in the reduced form of nicotinamide adenine dinucleotide (NADH) and a decrease in the NAD+/NADH ratio. This pathway results in the conversion of cytosolic xanthine dehydrogenase to xanthine oxidase, which is the enzyme responsible for generating superoxide radicals; NADH is also responsible for releasing iron (Fe) (II) ions from ferritin (Cederbaum et al. 2009). Free iron ions participate in the Haber–Weiss reaction, which ultimately leads to an increase in ROS generation (Nordmann et al. 1990).

In this study, we saw that the brains of ethanol-intoxicated rats show a significant decrease in the activity of antioxidant enzymes, including Cu,Zn-SOD, which is the principal enzyme that brain cells use to defend themselves from superoxide anion and, consequently, from other ROS (Liu et al. 2007). The decrease in superoxide dismutase activity may even exacerbate neuronal cell damage and death, as shown previously (Venkataraman et al. 2010; Scolaro et al. 2012). The hydrogen peroxide generated as a result of Cu,Zn-SOD action is removed by GSH-Px and catalase, but ethanol intoxication causes a decrease in their activity.

Changes in the activities of the above enzymes may result from molecular modifications caused by ROS generated during ethanol metabolism. The most reactive hydroxyl radical, generated during other ROS transformations, may nonspecifically oxidize all protein amino acid residues. Those residues most sensitive to oxidative modifications are aromatic residues, as well as residues containing sulfur (cysteine and methionine) (Huggins et al. 1993; Berlett et al. 1996). Moreover, the molecules of enzymatic proteins may be modified by the 1-hydroxyethyl radical generated during ethanol metabolism, which can decrease the activity levels of catalase and other enzymes (Puntarulo and Cederbaum 1989).

Administration of the solution of a natural plant—sweet grass—to ethanol-intoxicated rats prevented changes in the activity of the antioxidative enzymes examined in this study. Sweet grass contains coumarin derivatives, specifically 5,8-dihydroxycoumarin and 5-hydroxy-8-O-β-D-glucopyranosyl coumarin (Grigonis et al. 2005), which have proven antioxidative properties (Kostova 2006; Thuong et al. 2010; Slapšytė et al. 2013. Coumarin derivatives can inhibit the activity of xanthine oxidase (Nijveldt et al. 2001; Lin et al. 2008) and chelate transition metal ions (Kostova 2006), which may diminish the pro-oxidative action of ethanol. Hydroxyl derivatives of coumarins can also act as free radical scavengers, an action that is correlated with the number of hydroxyl groups in their structure (Lin et al. 2000; Huang et al. 2005). Thus, coumarins that possess hydroxyl groups are more potent free radical scavengers than their methoxy-substituted derivatives (Bilgin et al. 2011).

The rise in ROS levels, the disruption of the antioxidant system during ethanol intoxication, and the increased levels of free iron ions together lead to elevated oxidative stress and enhanced interactions between free radicals and lipids, which alter cellular structure and functions (Lu and Cederbaum 2008). Oxidative lipid metabolism is regulated mainly by GSH-Px, which quickly metabolizes lipid peroxides to less toxic hydroxyl derivatives (Rosenblat and Aviram 1998). However, GSH-Px activity depends on its co-substrate GSH, whose concentration is diminished after ethanol intoxication, as observed here. Disturbances in the GSH-Px system lead to increased lipid peroxidation. Enhanced lipid peroxidation results in increased levels of small molecular aldehydes, such as 4-hydroxynonenal, as well as the prostaglandin derivatives isoprostanes (8-isoPGF_2α_ and NPs. The last two compounds are the most stable and specific markers of lipid peroxidation and are superior to other markers of oxidative damage. 8-isoPGF_2α_ is formed mainly from peroxidation of AA, which is abundant in all types of cells, whereas NPs are derived from DHA, which is particularly enriched in neurons (Montuschi et al. 2004; Niki 2014).

Phospholipids of brain membranes are particularly vulnerable to free radical action because they contain large amounts of unsaturated fatty acids. These fatty acids can be peroxidized, which modifies their composition and fluidizes the phospholipid bilayer, thereby disturbing the functional state of the brain cells (Rottkamp et al. 2000). In the present study, we observed decreased brain levels of free and phospholipid AAs and DHAs, as well as significant increases in the brain levels of 8-isoPGF_2α_ and NPs after ethanol intoxication. The increase in the level of NPs was more significant than that of 8-isoPGF_2α_ which is a less specific marker of oxidative brain injury.

Changes in the above markers had the same direction and similar intensification as those observed in the brain levels of the small molecular aldehydes MDA and 4-hydroxynonenal after alcohol consumption, as observed here and previously (Skrzydlewska et al. 2007; Cyuńczyk et al. 2012). Both aldehydes are highly reactive and may act as “secondary toxic messengers” of the primary free radical events. However, the present results demonstrated that sweet grass given to rats intoxicated with ethanol prevented the increase in lipid peroxidation, as earlier proven under in vitro conditions (Thuong et al. 2010). This action of sweet grass could be connected with the improvements in the levels of lipophilic antioxidants, such as vitamins A and E, and decreases in ROS production, which prevent changes in membrane phospholipid disruption and protect biological membrane structure and functions, including membrane fluidity, as shown recently in vivo (Dobrzyńska et al. 2013).

Lipophilic antioxidants cooperate with GSH, but the concentration of this peptide is also diminished after ethanol intoxication. The GSH concentration depends on the levels of vitamins E and C because GSH is used, with the participation of vitamin C, during regeneration of vitamin E from tocopherol radicals (Bunker 1992). Drinking sweet grass solution increased GSH and ascorbate levels and protected them from ethanol action. Coumarin and its hydroxyl derivatives have previously been shown to protect the kidney from reduced GSH levels, leading to increased GSH-Px activity and nonenzymatic antioxidant defenses (Lake et al. 2002; Khan et al. 2004). Sweet grass consumption has been suggested to enhance GSH and ascorbate antioxidant action and/or to act synergistically to facilitate radical transfer o GSH and ascorbate (Winterbourn 2008).

In conclusion, given that the metabolism of ethanol and coumarins is similar in rats and humans, the results obtained in this paper suggest that sweet grass may also protect human brain cells against the consequences of oxidative stress induced by ethanol intoxication. Moreover, substantial and growing evidence for the bioactivity of sweet grass in vivo demonstrates its potential role in protecting human health and preventing the deleterious consequences of brain diseases associated with oxidative stress.
